# Lessons from a patient with cardiac arrest due to massive pulmonary embolism as the initial presentation of Wilms tumor: a case report and literature review

**DOI:** 10.1186/s12887-019-1413-y

**Published:** 2019-01-31

**Authors:** Atsuna Fukuda, Takeshi Isoda, Naoya Sakamoto, Keisuke Nakajima, Tetsuya Ohta

**Affiliations:** 1 Department of Pediatrics, JA Toride Medical Center, 2-1-1, Hongo, Toride, Ibaraki, Japan; 2 Department of Pediatrics and Developmental Biology, Tokyo Medical and Dental University, 1-5-45, Bunkyo-ku, Tokyo, Japan; 3 Department of Pediatric Surgery, JA Toride Medical Center, 2-1-1, Hongo, Toride, Ibaraki, Japan

**Keywords:** Case report, Wilms tumor, Massive pulmonary tumor embolism, Cardiac arrest

## Abstract

**Background:**

Finding an abdominal mass or hematuria is the initial step in diagnosing Wilms tumor. As the first manifestation of Wilms tumor, it is exceedingly rare for pulmonary tumor embolism to present with cardiac arrest. A case of a patient whose sudden cardiac arrest due to massive pulmonary tumor embolism of Wilms tumor was not responsive to resuscitation is presented.

**Case presentation:**

The patient was a five-year-old girl who collapsed suddenly during activity in nursery school and went into cardiac arrest in the ambulance. Unfortunately, she was not responsive to conventional resuscitation. A judicial autopsy conducted at the local police department showed the main cause of her sudden cardiac arrest was attributed to multiple pulmonary tumor embolisms of stage IV Wilms tumor.

**Conclusions:**

Except for one reported case, treatments were not successful in all eight cardiac arrest cases with pulmonary tumor embolism of Wilms tumor. These results indicate that it is challenging not only to make an accurate diagnosis, but also to provide proper specific treatment in the cardiac arrest setting. We propose that flexible triage and prompt transfer to a tertiary hospital are necessary as an oncologic emergency to get such patients to bridging therapy combined with extracorporeal membrane oxygenation or immediate surgical intervention under cardiopulmonary bypass.

## Background

Massive pulmonary tumor embolism of Wilms tumor is a rare condition that often leads to fatal outcomes. Invasion of Wilms tumor into the inferior vena cava (IVC) and right atrium is well known. The National Wilms’ Tumor Study (NWTS), UK Children’s Cancer Study Group (UKW) and the International Society of Paediatric Oncology (SIOP) studies reported that the rates of pre-operative thrombosis in the IVC and heart were roughly 5% and less than 1%, respectively [1–3]. Although intravascular Wilms tumor with anaplastic histology and clear cell sarcoma have less favorable prognoses, relapse-free survival of Wilms tumor with favorable histology is comparable between intravascular and non-intravascular tumors [2]. Including the present patient, nine cases of sudden unexpected cardiac arrests due to massive pulmonary tumor embolism of Wilms tumor have been reported (Table 1) [4–9]. Massive pulmonary embolism was not associated with a specific histological subtype of Wilms tumor. The case of a patient with massive pulmonary tumor embolism of Wilms tumor who suddenly collapsed and developed cardiac arrest in an emergency vehicle is reported. Based on our experience, we propose that a patient with a massive pulmonary tumor embolism of Wilms tumor requires prompt transfer to a facility that can provide resuscitation combined with extracorporeal membrane oxygenation (ECMO) or immediate surgical intervention with cardiopulmonary bypass. This strategy would be the sole potentially curative pathway to remove pulmonary tumor embolism by surgical resection as a bridge to conventional therapy for this invasive type of Wilms tumor.

**Table 1:**
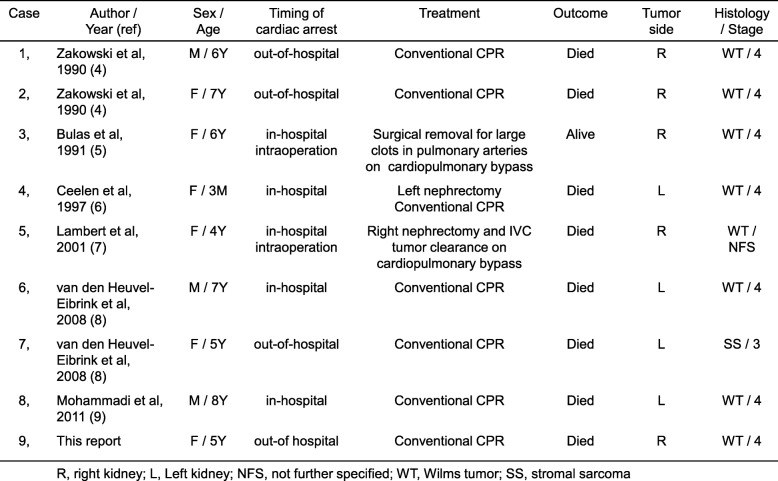
Table 1

### Case presentation

**Fig. 1:**
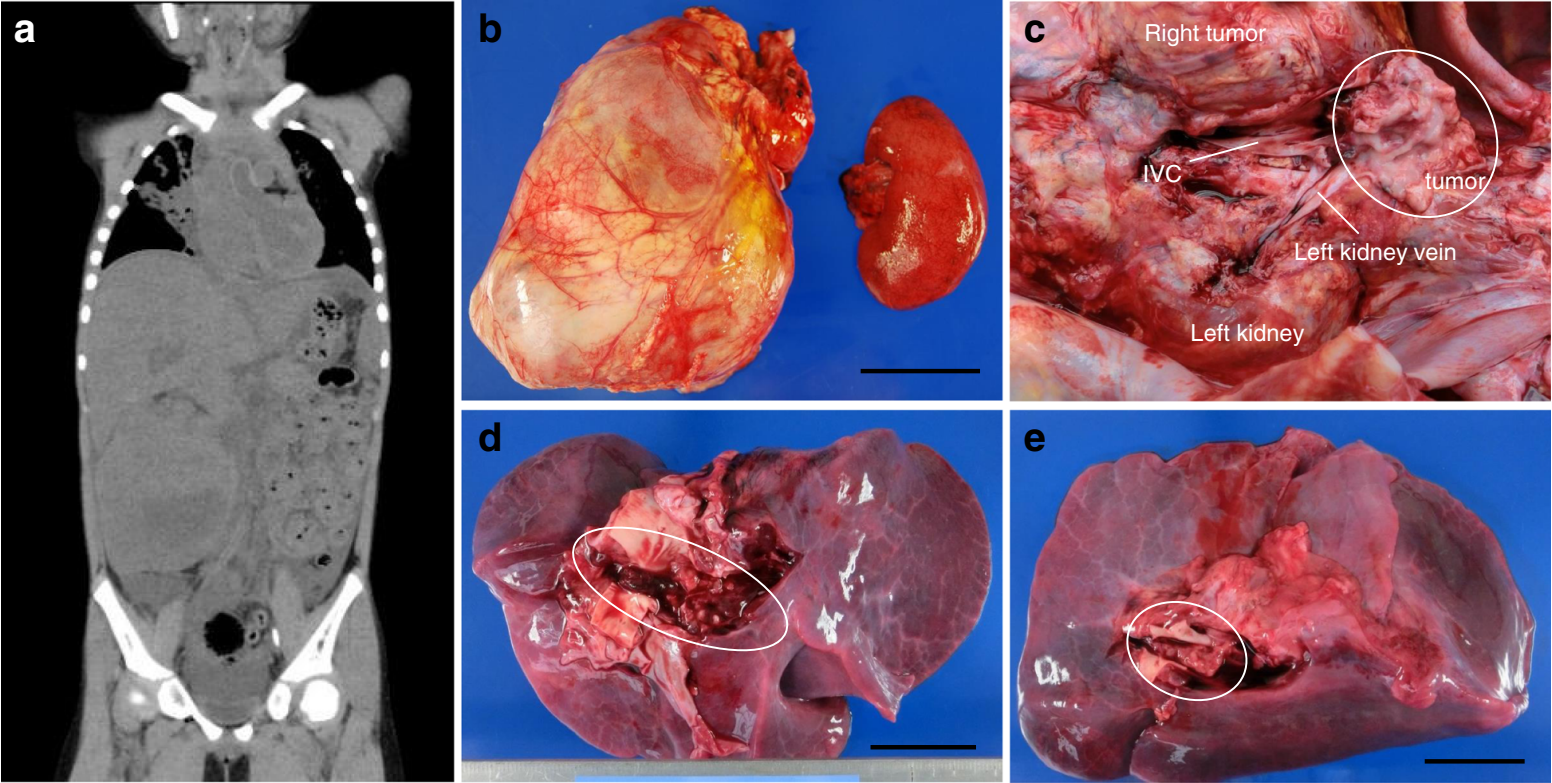
**a** Plain postmortem CT scan shows a right tumor mass extending into the inferior vena cava (IVC). **b** Right tumor and normal left kidney are shown. **c** The tumor extends into the IVC. **d** Multiple greyish tumors and blood clots are observed in the right pulmonary artery. **e** Greyish tumor occupies the left pulmonary artery. Black bar represents 5 cm

The patient was a five-year-old girl who collapsed suddenly during activities at nursery school. She was healthy until that day. She had passed a regular health check one month before the event. Regional emergency workers transferred her to our hospital located 30 min away from the event and categorized as a secondary healthcare hospital without extracorporeal cardiopulmonary resuscitation (ECPR) capability. Her condition deteriorated, and she developed cardiac arrest in the ambulance. Regional emergency workers commenced Basic Life Support (BLS) while transferring her to the emergency room. Resuscitation with Advanced Life Support including intubation and repeated epinephrine was given to her after arrival at the emergency department (ED). Unfortunately, after resuscitation for a total of 81 min inclusive of four minutes pre-hospital BLS, she remained unresponsive; the resuscitation was unsuccessful. Venous blood gases on arrival to the ED showed *p*CO_2_ 65.8 mmHg, and *p*O_2_ 29.7 mmHg. pH, bicarbonate, and base excess were immeasurable possibly due to out of range of indication. Blood tests showed the following abnormal values: prothrombin time 20.2 s; APTT 88.7 s; D-dimer 106.3 μg/ml; FDP 249.8 μg/ml; potassium 7.3 mEq/L; creatinine 0.71 mg/dL; AST 65 U/l; LDH 821 U/l; and ammonia 477 μg/ml. Postmortem CT showed a large right abdominal mass extending through the IVC into the entry portion of the right atrium (Fig. 1a).

**Fig. 2:**
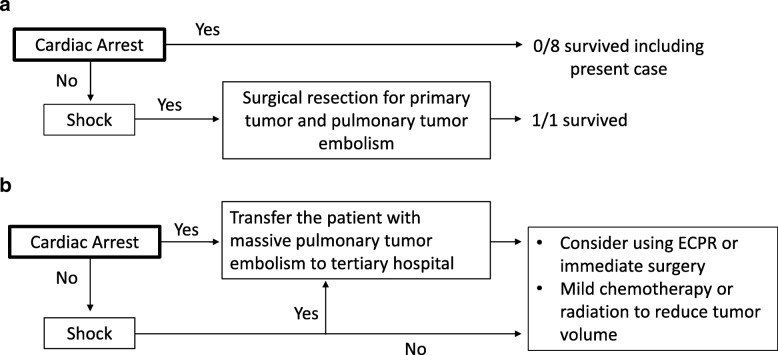
**a** Outcomes of Wilms tumor with massive pulmonary embolism (PE) in past reports and the present case. **b** Proposed strategy for Wilms tumor with massive PE and cardiac arrest or shock. ECPR, extracorporeal cardiopulmonary resuscitation; PH, pulmonary hypertension; RV, right ventricle

A judicial autopsy conducted at the local police department showed: [1] the weight of the Wilms tumor that originated in the right kidney was 885 g, while the left kidney weighed 100 g, and no further histological examination was performed (Fig. 1b); [2] tumor extended into the right renal vein, IVC, and entry portion of the right atrium (Fig. 1c); and [3] greyish or dark red small multiple emboli filled the right and left peripheral pulmonary arteries (Fig. 1d and e). Taken together, the main cause of her sudden cardiac arrest was attributed to multiple pulmonary tumor embolisms secondary to stage IV Wilms tumor.

## Discussion and conclusions

Massive pulmonary tumor embolism consists of the tumor itself, and secondary thrombus is a rare fatal condition in Wilms tumor. Similar to past reports, the present case was unresponsive to conventional resuscitation while being transferred in an emergency vehicle. Including the present patient, nine pediatric cases of sudden unexpected cardiac arrests due to massive pulmonary tumor embolism of Wilms tumor have been reported (Table 1) [4–9]. However, there could be reporting bias, and the actual incidence may be higher because autopsies in cases of sudden death were not always performed, or confirmed cases were simply not reported. In our view, the only chance for an earlier diagnosis would have been the detection of an abdominal mass in the patient one month before this event. Given this case, all physicians should carefully perform abdominal palpation, since, except for one case, treatments were not successful in all other cardiac arrest cases with massive pulmonary tumor embolism of Wilms tumor (Fig. 2a)(Table 1), indicating that it can be challenging not only to make an accurate diagnosis, but also to provide proper specific treatment in the cardiac arrest setting. All cases including the present case showed sudden collapse and cardiac arrest [4–10]. The one patient who survived was alive at the hospital when diagnosed with massive pulmonary tumor embolism of Wilms tumor [5]. This patient suddenly went into cardiac arrest during surgery but was resuscitated and placed on cardiopulmonary bypass. The large clots from the main and right pulmonary arteries and the original right kidney tumor with IVC thrombus were successfully removed [5]. Although there has been a limited number of children with pulmonary embolism (PE), compared to PE due to coagulation disorder, pulmonary tumor embolism in pediatric cancer invasion showed much worse outcomes [11].

Non-neonatal pediatric ECMO cases with a variety of indications have increased [12]. In the past tumor-related reports, ECPR was performed for a patient with metastatic choriocarcinoma, a patient with lymphoma, and another patient with leukemia who developed PE [13–15]. The case with choriocarcinoma presented with severe dyspnea, massive hemoptysis, and decreased mean BP in the hospital. Venoarterial (VA) ECMO was used to restore hemodynamic stability. Subsequently, the patient was successfully cured with a pulmonary embolectomy and chemotherapy under ECMO [13]. Although the third case was not successfully resuscitated even with ECMO support and emergency surgery in the hospital [14], both cases were started on ECMO before cardiac arrest. A patient with lymphoma who had a cardiac arrest for 8 min and achieved successful recovery of spontaneous circulation received bridging ECMO support, leading to discharge without any other serious complications [15]. We suggest that ECMO at tertiary hospitals should be considered for potentially fatal cases of massive pulmonary tumor embolism of Wilms tumor before or immediately after cardiac arrest (Fig. 2b) [16–18]. Also, it is important to note that the femoral route as access for ECPR should be avoided in a patient with massive pulmonary tumor embolism because of infiltration of Wilms tumor into the IVC. Thus, V-A ECMO draining via the internal jugular venous cannula or immediate surgical intervention under cardiopulmonary bypass might have been a potential option for the present patient.

Some centers have developed PE teams involving hematology, ED staff, intensive care staff, cardiologists, and pediatric surgeons [19]. Prompt treatment by the in-house multidisciplinary team is essential for the treatment of similar cases as an oncologic emergency [20]. We also expect that development of immediate onsite triage combined with portable echocardiogram would provide a better strategy for initial selection and set up for potential use of ECPR systems [15, 21–23] (Fig. 2b). Ketelaars et al. showed that prehospital chest ultrasound on an air emergency medical service can flexibly alter the destination and improve treatment decisions for adult patients [24]. Based on our experience, we suggest that the destination decision to a tertiary hospital and a prompt transfer system would be a critical first step for transporting the patient to an ECPR center or immediate surgery in a patient with fatal massive pulmonary tumor embolism of Wilms tumor (Fig. 2b). Further experience will be needed to determine how best to get a patient with this type of oncologic emergency to conventional therapy.

## Abbreviations

ECMO: Extracorporeal membrane oxygenation
ECPR: Extracorporeal cardiopulmonary resuscitation
ED: Emergency department
PE: Pulmonary embolism
